# Improved genetically-encoded, FlincG-type fluorescent biosensors for neural cGMP imaging

**DOI:** 10.3389/fnmol.2013.00026

**Published:** 2013-09-24

**Authors:** Yogesh Bhargava, Kathryn Hampden-Smith, Konstantina Chachlaki, Katherine C. Wood, Jeffrey Vernon, Charles K. Allerston, Andrew M. Batchelor, John Garthwaite

**Affiliations:** ^1^Wolfson Institute for Biomedical Research, University College LondonLondon, UK; ^2^Structural Genomics Consortium, University of OxfordOxford, UK

**Keywords:** cGMP, genetically-encoded biosensor, nitric oxide, neuroblastoma, hippocampus, dorsal root ganglion, C-type natriuretic peptide

## Abstract

Genetically-encoded biosensors are powerful tools for understanding cellular signal transduction mechanisms. In aiming to investigate cGMP signaling in neurones using the EGFP-based fluorescent biosensor, FlincG (fluorescent indicator for cGMP), we encountered weak or non-existent fluorescence after attempted transfection with plasmid DNA, even in HEK293T cells. Adenoviral infection of HEK293T cells with FlincG, however, had previously proved successful. Both constructs were found to harbor a mutation in the EGFP domain and had a tail of 17 amino acids at the C-terminus that differed from the published sequence. These discrepancies were systematically examined, together with mutations found beneficial for the related GCaMP family of Ca^2+^ biosensors, in a HEK293T cell line stably expressing both nitric oxide (NO)-activated guanylyl cyclase and phosphodiesterase-5. Restoring the mutated amino acid improved basal fluorescence whereas additional restoration of the correct C-terminal tail resulted in poor cGMP sensing as assessed by superfusion of either 8-bromo-cGMP or NO. Ultimately, two improved FlincGs were identified: one (FlincG2) had the divergent tail and gave moderate basal fluorescence and cGMP response amplitude and the other (FlincG3) had the correct tail, a GCaMP-like mutation in the EGFP region and an N-terminal tag, and was superior in both respects. All variants tested were strongly influenced by pH over the physiological range, in common with other EGFP-based biosensors. Purified FlincG3 protein exhibited a lower cGMP affinity (0.89 μM) than reported for the original FlincG (0.17 μM) but retained rapid kinetics and a 230-fold selectivity over cAMP. Successful expression of FlincG2 or FlincG3 in differentiated N1E-115 neuroblastoma cells and in primary cultures of hippocampal and dorsal root ganglion cells commends them for real-time imaging of cGMP dynamics in neural (and other) cells, and in their subcellular specializations.

## Introduction

The development of relatively non-invasive, genetically-encoded detectors that allow real-time imaging of second messengers, such as Ca^2+^ and cyclic nucleotides, in living cells has greatly advanced the understanding of cellular signal transduction (Tsien, 1998; Mehta and Zhang, 2011). One cyclic nucleotide, cGMP, is found in almost all cells where it serves as the second messenger for nitric oxide (NO) and/or for peptide transmitters, including the natriuretic peptides. cGMP can engage multiple downstream pathways, including cGMP-dependent protein kinase (PKG), to bring about acute or long-term alterations in cellular function. A number of cGMP biosensors that exploit Förster resonance energy transfer have been engineered (Vincent et al., 2008; Thunemann et al., 2013) but a particularly promising approach has capitalized on the success of the GCaMP family of Ca^2+^-sensitive, single fluorescent proteins in which Ca^2+^-binding domains are fused to circularly permuted EGFP (cpEGFP) (Nakai et al., 2001; Zhao et al., 2011; Akerboom et al., 2012). The analogous cGMP biosensor, called FlincG (standing for fluorescent indicator for cGMP; specifically the δ-version; Nausch et al., 2008), incorporates a cGMP-binding domain of PKG linked to cpEGFP. It shows 100-fold or more selectivity over cAMP, is sensitive to submicromolar concentrations of cGMP and displays rapid kinetics, allowing a faithful readout of dynamic changes in cGMP within the physiological range (Nausch et al., 2008; Batchelor et al., 2010; Wood et al., 2011). Moreover, unlike the GCaMP series whose fluorescence is strongly influenced by pH (Nakai et al., 2001; Wang et al., 2008; Zhao et al., 2011), FlincG is reported to be resistant to small pH changes by virtue of having a low pK_a_ of 6.1 (Nausch et al., 2008).

Thus far, FlincG has been expressed in mammalian cells mainly through the use of a serotype-5 adenoviral vector. This method of delivery has proved successful in smooth muscle cells, HEK293T cells and cardiac fibroblasts (Nausch et al., 2008; Batchelor et al., 2010; Miller et al., 2011; Wood et al., 2011) but it is unsuited to many other cell types of interest, such as neurones, which lack the coxsackie and adenovirus receptor needed to facilitate internalization (e.g., Lewis et al., 2010). A plasmid (pcDNA3.1) containing a δ-FlincG clone was provided to us by the originator laboratory but, in attempts to transfect HEK293T cells or primary neuronal cultures, little or no fluorescence above background could be detected, suggesting a low level of FlincG protein expression and/or lack of protein fluorescence.

In seeking an explanation for the poor performance of the cGMP biosensor with conventional cDNA transfection compared to adenoviral infection, we rescued the FlincG DNA from the adenoviral genome and found disparities in both the plasmid and adenoviral DNA compared with the published sequence. The present report examines the importance of rectifying these disparities for obtaining a usable FlincG plasmid and further investigates the potential benefit of mutations in the cpEGFP region that increased the baseline fluorescence and dynamic range of GCaMP biosensors, as well as other sequence modifications.

## Materials and methods

### Materials

1H-[1,2,4]Oxadiazolo[4,3-*a*]quinoxalin-1-one (ODQ) and carbonyl cyanide *m*-chlorophenylhydrazone (CCCP) were from Tocris Bioscience; 2-(4-carboxyphenyl)-4,4,5,5-tetramethylimidazoline-1-oxyl-3-oxide (CPTIO), 1-hydroxy-2-oxo-3-(N-ethyl-2-aminoethyl)-3-ethyl-1-triazene (NOC-12), 8-bromo-cGMP, N^G^-nitro-L-arginine, Texas Red, proteinase-K, nigericin, nerve growth factor, collagenase type XI, dispase and superoxide dismutase were from Sigma-Aldrich; fluorescein was from Millipore; (Z)-1-[N-(3-ammoniopropyl)-N-(n-propyl)amino]diazen-1-ium-1,2-diolate] (PAPA/NO) was from Enzo Life Sciences; FuGene and Lipofectamine were from Promega and Invitrogen, respectively. Common laboratory chemicals were from Sigma-Aldrich. Stock solutions of NOC-12 and PAPA/NO were made up in 10 mM NaOH and kept on ice; they were diluted 1000-fold into the experimental solution.

### Rescuing FlincG DNA from pAd-DEST adenoviral vector

The generation of pAd-DEST adenoviral vector containing FlincG was described elsewhere (Nausch et al., 2008). This vector was isolated from adenoviral particles using the method of Le et al. (2001). Briefly, adenoviral particles in tris-buffered saline were treated with 1% SDS in the presence of proteinase-K. Vector DNA was precipitated using ethanol after phenol-chloroform extraction. To determine the sequence of adenoviral FlincG (AdV-FlincG) DNA, it was subcloned from the recombinant adenoviral genome into the pENTR1a vector using BP clonase (Invitrogen) as per the manufacturer's protocol. Briefly, 150 ng each of adenoviral DNA and pENTR1a were mixed with 2 μl of BP clonase and incubated at room temperature for 90 min. The reaction was treated with 20 μg/ml proteinase-K and the DNA purified using a Qiaquick column (Qiagen). The pENTR1a containing AdV-FlincG DNA was propagated in *E. coli* DH5α cells selected for kanamycin antibiotic resistance. The clones were verified by DNA sequencing.

### Generation of FlincG mutants

**Figure 1:**
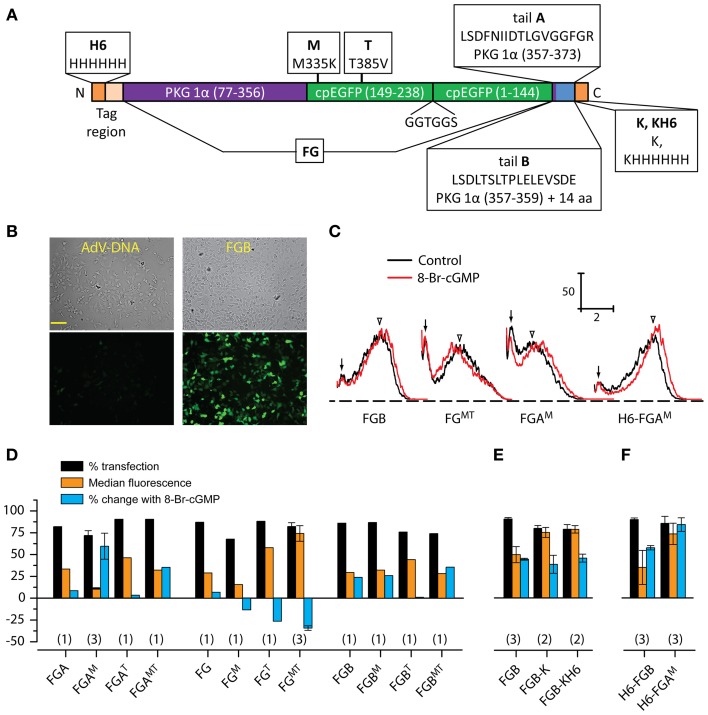
**Screening of FlincG variants. (A)** Schematic diagram illustrating the general FlincG design and specifying the modifications tested. The diagram also defines the nomenclature adopted in text. **(B)** Brightfield (top) and basal fluorescence (bottom) images of HEK_GC/PDE5_ cells 3 days after attempted transfection with identical amounts (0.5 μg/well) of AdV-FlincG plasmid DNA (left) and FGB DNA (right). Scale bar = 100 μm. **(C)** Sample FACS spectra of suspensions of HEK_GC/PDE5_ cells transfected with the indicated FlincG variants, before (black) and after (red) incubation with 8-bromo-cGMP (1 mM). Calibration bars: vertical = frequency; horizontal = log intensity (arbitrary units). Arrows and arrowheads indicate untransfected and transfected components of the control spectra, respectively. **(D–F)** Results of FACS analysis for groups of FlincG variants; plasmids in panel D all bore a strong Kozak consensus sequence (see Material and Methods). Numbers in parentheses are *n*-values.

The AdV-FlincG DNA was located between BamHI and EcoRI restriction sites. The DNA sequence between these two sites had a partial Kozak sequence (ACCATGG) followed by the PKG and cpEGFP regions, ending with a 17-amino acid “tail region” (Figure 1A). The originating laboratory provided the same cDNA sequence subcloned into the plasmid pcDNA3.1 (Nausch et al., 2008). Approximately 850 surplus nucleotides beyond the stop codon in both clones (coding for the catalytic domain of PKG) were deleted in all variants included in this study, although in a preliminary test with one variant (called FGB below), the truncation made no obvious difference to its performance in imaging experiments. Various point mutants containing tail A, tail B or no tail were generated using either the overlapping primer extension method described by Liu and Naismith (2008) or using the QuikChange II site-directed mutagenesis kit (Agilent Technology Inc.) according to the manufacturer's protocol. For cloning into expression vectors, we used two plasmids, namely the pEGFP-C1 vector (Clontech) with either a partial (ACCATGGCA) or full (GCCACCATGGTA) Kozak consensus sequence, and the pTriEx-4 vector which has the above partial Kozak sequence (Merck Millipore). The 3′-terminal triplet of each Kozak sequence codes for the second amino acid, which is alanine or valine (Figure S1), the former being in the original adenoviral and plasmid-based clones. As AdV-FlincG DNA gave poor basal fluorescence (see Results), we initially speculated that this alanine might promote protein degradation (Gonda et al., 1989) following normal post-translational hydrolysis of the methionine in the first position and so it was substituted for valine. Point mutations are denoted in the text with a superscripted suffix indicating the single-letter code of the mutated amino acid (see Figure 1). All mutations were verified by DNA sequencing using primers listed in Supplementary Table 1.

### Protein purification

One-liter cultures of Terrific Broth (Novagen), supplemented with 4 ml glycerol, 50 μg/ml ampicillin and 50 μg/ml chloramphenicol were prepared in UltraYield baffled flasks. The flasks were inoculated with 10 ml of an overnight culture of BL21 (DE3) cells harboring the pTriEx-4 vector with an N-terminal hexahistidine-tagged FlincG variant. Cultures were grown at 37°C with shaking at 250 rpm. Induction was carried out at an OD_600_ of ~3.5 with 0.5 mM isopropylthio-β-galactoside and the cultures were further incubated at 18°C overnight at 250 rpm. The next day, cells were harvested by centrifugation at 4°C and the pellets resuspended in two volumes per unit weight of lysis buffer (50 mM tris, 500 mM NaCl, 5% glycerol, 5 mM imidazole, 0.5 mM tris(2-carboxyethyl)phosphine hydrochloride, pH 8.0). Cells were lysed by sonic disruption and nucleic acids and cell debris removed by adding polyethyleneimine to a final concentration of 0.15%. The solution was clarified by centrifugation at 4°C for 1 h at 25,000 g. Immobilized metal affinity chromatography using a 5 ml His-trap column (GE Healthcare) employed the above-mentioned lysis buffer, supplemented with 30 mM imidazole. Bound protein was eluted in the same buffer with an increased imidazole concentration (300 mM). Finally, the eluted protein was subjected to gel filtration on a Superdex 200 column (GE Healthcare) in 25 mM tris (pH 8.0), 0.1 M NaCl, 1 mM tris(2-carboxyethyl)phosphine hydrochloride and 5% glycerol. Monomeric peak samples eluted from the gel were pooled and concentrated to 25–30 μM using Amicon spin filters (Ultracel PL-10, 10,000 mW cut-off; Millipore). Protein was assayed using the Bradford method. The concentrated protein was snap-frozen in liquid nitrogen and stored at −80°C until use.

### Spectroscopy

Excitation (emission at 510 nm) and emission spectra (excitation at 490 nm) of purified N-terminal hexahistidine-tagged FlincG proteins (10–30 nM) were recorded at room temperature through the 1-cm light path of a 4-window, 1.5-ml quartz cuvette using the Fluoromax-3 spectrofluorometer (Horiba Inc). To measure cyclic nucleotide sensitivity, purified protein was added in a BHEK-150 buffer containing BSA (0.3 mg/ml), HEPES (30 mM), EDTA (20 μM) and KCl (150 mM), pH 7.4 (at room temperature), osmolality 290–300 mosmol/kg. Inclusion of BSA and EDTA in the buffer at the stated concentrations prevented artifactual spectral changes during mixing; presumably, BSA blocked non-specific protein interactions and EDTA chelated any free nickel or other contaminating metal ions. Increasing concentrations of cGMP, cAMP, or 8-Br-cGMP were added incrementally to generate concentration-response curves, the total ligand increasing the volume by less than 1%. The mixtures were stirred magnetically for more than 1 min prior to spectral measurements. For testing pH sensitivity, a pH titration buffer containing tri-sodium citrate (30 mM) and Tris-Cl (30 mM) was used, its pH being adjusted from 9 to 5 (at 0.5 intervals) using HCl at room temperature. After addition of BSA (0.3 mg/ml), EDTA (20 μM) and FlincG protein (20–30 nM), fluorescence spectra were recorded before and after addition of 100 μM cGMP.

### Cell culture and transfection

HEK293T cells constitutively expressing NO-activated guanylyl cyclase and phosphodiesterase-5 were provided by Professor Doris Koesling (University of Bochum, Germany). These cells have been extensively characterized in previous studies (Mullershausen et al., 2004; Batchelor et al., 2010; Wood et al., 2011) and are referred to here as HEK_GC/PDE5_ cells. They were maintained and transfected as described (Batchelor et al., 2010).

N1E-115 neuroblastoma cells (ATCC, Teddington, Middlesex, UK) were cultured according to the supplier's protocol in a growth medium consisting of Dulbecco's modified Eagle's medium (DMEM) containing L-glutamine and 4.5 g/l glucose (Invitrogen) supplemented with NaHCO_3_ (1.5 g/l), fetal bovine serum (10%) and penicillin/streptomycin (100 U/ml and 100 μg/ml, respectively), in a humidified, 5% CO_2_ atmosphere. The cells were split at 70% confluency and the medium changed every 2–3 days. For imaging, cells were plated onto poly-D-lysine-coated glass coverslips in 24-well plates and were differentiated by transfer into growth medium with 2% fetal bovine serum and 1.25% dimethylsulfoxide (Oh et al., 2005) for 3–4 days prior to transfection using 1 μg DNA/well and FuGene 6 (6 μl/μg DNA). Transfection was aided by briefly (4 min) centrifuging the culture plates (128 g) at room temperature. The cultures were then maintained for 3 days in growth medium lacking antibiotics and containing 1% fetal bovine serum and 0.625% dimethylsulfoxide, and for a further 3 days in normal differentiation medium, before being used in experiments.

All animal use was approved by the local (UCL) ethics committee and was carried out strictly in accordance with the UK Animals (Scientific Procedures) Act 1986. Mixed neurones from newborn rat hippocampi were plated onto a feeder layer of astrocytes in 24-well plates according to published methods (Huettner and Baughman, 1986; Morales et al., 2000). After 7–10 days the medium was changed to an astrocyte-conditioned medium lacking antibiotics and transfection (0.5 μg DNA per well) was carried out using FuGene 6 (3 μl/μg DNA), facilitated by a brief centrifugation (4 min at 128 g) at room temperature. The cultures were used for imaging 2–3 days later.

Dorsal root ganglion cells were isolated and cultured using standard methods (Rugiero et al., 2010) with minor alterations. Briefly, the ganglia were removed from 8-day-old rats and digested in collagenase type XI (2.5 mg/ml), dispase (10 mg/ml) and glucose (10 mM) in Hank's balanced salt solution (Invitrogen) for 25 min at 37°C in 5% CO_2_. After the addition of DMEM containing 10% fetal bovine serum to stop the digestion, cells were dissociated by trituration, collected by low-speed centrifugation, and resuspended in antibiotic-free medium composed of DMEM containing 1% glutamax, 10% fetal bovine serum and 125 ng/ml nerve growth factor and then plated onto glass coverslips pre-coated with poly-D-lysine (0.5 mg/ml) and laminin (0.02 mg/ml). After allowing 30–60 min at 37°C for attachment, the cells were transfected (0.5–1 μg DNA per well) with either FuGene 6 (3 μl/μg DNA) or Lipofectamine 2000 (1 μl/μg DNA). Plates were centrifuged (4 min, 128 g) at room temperature to aid transfection and were then incubated at 37°C in 5% CO_2_ for 2–3 days before use.

### Fluorescence-activated cell sorter (FACS) analysis

HEK_GC/PDE5_ cells were transfected with 0.5 μg DNA and 3 μl FuGene 6 per well, seeded into 12-well plates and cultured for 3 days, by which time they had reached confluency. The cells were collected by trypsinization and resuspended into the buffer used for cell imaging (see below). FACS analysis was performed using a CyAn ADP High-Performance Flow Cytometer (Beckman Coulter) equipped with a GFP filter (488 nm laser-line excitation). A total of 12,000 gated events were sampled before and after stimulation with 8-bromo-cGMP (1 mM, 37°C, 10 min preincubation). The same gates, set to exclude debris, dead cells and cell clumps, were used throughout. The frequency-fluorescence spectra displayed two distinct but overlapping peaks, representing populations of untransfected and transfected cells (see Results). The peaks were resolved by fitting the spectra in Mathcad 14 (Parametric Technology Corporation) to the sum of two asymmetric double-sigmoidal functions of the following type:

$$y=\left[A\left[\frac{1}{1\text{​}+\text{​exp}\left(\frac{-\left(x-xc+\frac{w1}{2}\right)}{w2}\right)}\right]\left[1-\frac{1}{1\text{​}+\text{​exp}\left(\frac{-\left(x-xc-\frac{w1}{2}\right)}{w3}\right)}\right]\right]$$

where *A* is the amplitude, *xc* the center, and *w*1, *w*2 and *w*3 are widths. Values of the parameters were obtained by minimizing the sum of the squares of the errors between data and fit, using the “Minerr” function in Mathcad. Quantification of the peaks and of the shifts brought about by 8-bromo-cGMP were carried out as illustrated in the accompanying annotated Mathcad worksheet (Supplementary Methods).

### Live cell imaging

Imaging was conducted using an inverted microscope as described (Batchelor et al., 2010), with minor modifications. In brief, glass coverslips with adhering cells were held in a chamber (0.5 ml volume) that was superfused continuously (1.5 ml/min) with warm (37°C) solution containing: NaCl (136 mM), KCl (2 mM), MgSO_4_ (1.2 mM), KH_2_PO_4_ (1.2 mM), CaCl_2_ (1.5 mM), glucose (5.5 mM) and HEPES (10 mM), pH 7.4. For delivery of clamped NO concentrations, N^G^-nitro-L-arginine (30 μM), superoxide dismutase (100 U/ml), CPTIO (0.1 mM) and urate (10 μM for murine cells; 100 μM for HEK_GC/PDE5_ cells), were also included. With CPTIO and urate present, addition of the slow NO releaser NOC-12 (half-life = 100 min) produces fixed NO concentrations that are proportional to the donor concentration (Griffiths et al., 2003). NO was applied in this way either by superfusion or from a nearby puffer pipette. With delivery by superfusion, access of NO to the cells was quantified by superfusing fluorescein (1 nM) at the end of the experiment. For puffer application, Texas Red dye (10 μM) was included in the pipette solution and the NO concentration determined by dividing the fluorescence intensity of the dye over the cell of interest by the peak intensity found just outside the mouth of the pipette, which was taken to be the same as the pipette concentration (1 nM NO). Epifluorescent signals were captured by camera, corrected for background, and displayed as the change in intensity relative to baseline divided by the baseline intensity (Δ*F*/*F*_0_).

### Statistics and curve fitting

Values are presented as means ± SEM; *n*-values represent numbers of independent experiments. Parameters describing concentration-response curves together with their standard errors were obtained from fits to the logistic equation in Origin 8.6 (OriginLab); pH parameters were derived from fits of titration curves (in Origin 8.6) to the following equation (Kneen et al., 1998):

$$F=A+\frac{B}{1+10^{n_{\text{H}}({\text{pK}}_{a}\prime -\text{pH})}}$$

where *F* = fluorescence or change in fluorescence (Δ*F*/*F*_0_), *n*_*H* = slope_ of the curve, pK_*a*_' = apparent pK_*a*_, *A* is the baseline of the curve and B, the maximum signal above baseline.

## Results

### FlincG engineering

DNA sequencing revealed that the adenoviral and plasmid-based FlincGs were both point mutated (R349C; numbering as in AdV-FlincG DNA; Figure S1) in the cpEGFP region. Also common to both sequences, the codons of 17 amino acids in the “tail-region” represented a sequence that mostly differed from the published sequence. We call the tail of the published sequence “tail A” and the divergent one “tail B” (Figure 1A). The effect of removing the entire tail region was also examined. On top of these modifications, we wished to test if incorporating one or both of two mutations in the cpEGFP domain that led to improvements in a related GCaMP Ca^2+^ biosensor (Tian et al., 2009) have a similarly beneficial effect on FlincGs. Accordingly, a matrix of alterations to the tail region and the cpEGFP domain was generated and systematically tested using a HEK293T cell line stably expressing NO-activated guanylyl cyclase and phosphodiesterase-5 (HEK_GC/PDE5_ cells; Mullershausen et al., 2004) as a model.

Restoring the arginine at position 349 led to an obvious improvement in the fluorescence of cells transfected with the FlincG clone containing tail B (Figure 1B) and so all further modifications retained this correction. The effects of variations of the tail and/or cpEGFP regions were first screened using FACS analysis of transfected HEK_GC/PDE5_ cells in the absence and presence of the membrane-permeating cGMP analogue, 8-bromo-cGMP. The frequency-fluorescence intensity distributions displayed two components (Figure 1C). One component (arrows, Figure 1C) had a similarly low intensity regardless of the cDNA used for transfection and the other (open arrowheads, Figure 1C) had a higher, but more variable, fluorescence. The relative fluorescence intensity of the two populations also varied with different FlincGs, with only the broader, brighter population shifting in the presence of 8-bromo-cGMP. Sham-transfected cells (*n* = 3) could be fitted to a single population that coincided with the low-intensity component (*n* = 34): the median fluorescence values (in log arbitrary units) were, respectively, 0.17 ± 0.013 and 0.18 ± 0.004 in the absence of 8-bromo cGMP, and 0.15 ± 0.011 and 0.15 ± 0.007 in its presence. Consequently, the components having low 8-bromo cGMP-insensitive fluorescence and higher 8-bromo cGMP-sensitive fluorescence are taken to represent untransfected and transfected cell populations, respectively. Deconvolution of the FACS spectra enabled values for the % transfection, the median basal fluorescence and the shift in fluorescence brought about by 8-bromo cGMP to be quantified (see Materials and Methods).

None of the alterations substantially affected the transfection efficiency, which remained at around 80%. Correcting tail B to the published tail A, producing the variant FGA in the nomenclature explained in Figure 1A, resulted in only a small response to 8-bromo cGMP compared with the version with the uncorrected tail B (FGB, Figure 1D), as did complete removal of the tail (FG, Figure 1D). When the mutations of the cpEGFP region were imposed alone or in combination on these tail variants, no consistent pattern emerged. The most notable effect of the M-mutation (M335K) was on the behavior of FGA (giving FGA^M^): on the negative side, the basal fluorescence was low (see, for example, the relatively small overlap between transfected and untransfected components of the FACS spectrum in Figure 1C) but a positive effect was an increase in the amplitude of the response to 8-bromo-cGMP compared with either FGA or FGB. The same mutation in FGB had no obvious effect on either basal fluorescence or 8-bromo-cGMP biosensitivity. On its own, the T-mutation (T385V) appeared to inhibit biosensing by both FGA and FGB but with the tailless FG variant, it enhanced basal fluorescence. Moreover, in response to 8-bromo-cGMP, the FG^T^ fluorescence became reduced, rather than increased, an effect also seen to a lesser extent with the M-mutation (FG^M^). With the double mutant (FG^MT^), both basal fluorescence and the amplitude of the dimming in the presence of 8-bromo-cGMP appeared to be accentuated compared with the single T-mutation. Addition of the M-mutation also partially or completely compensated for the inhibitory effect of the T-mutation on the responsiveness of FGA and FGB.

**Figure 2:**
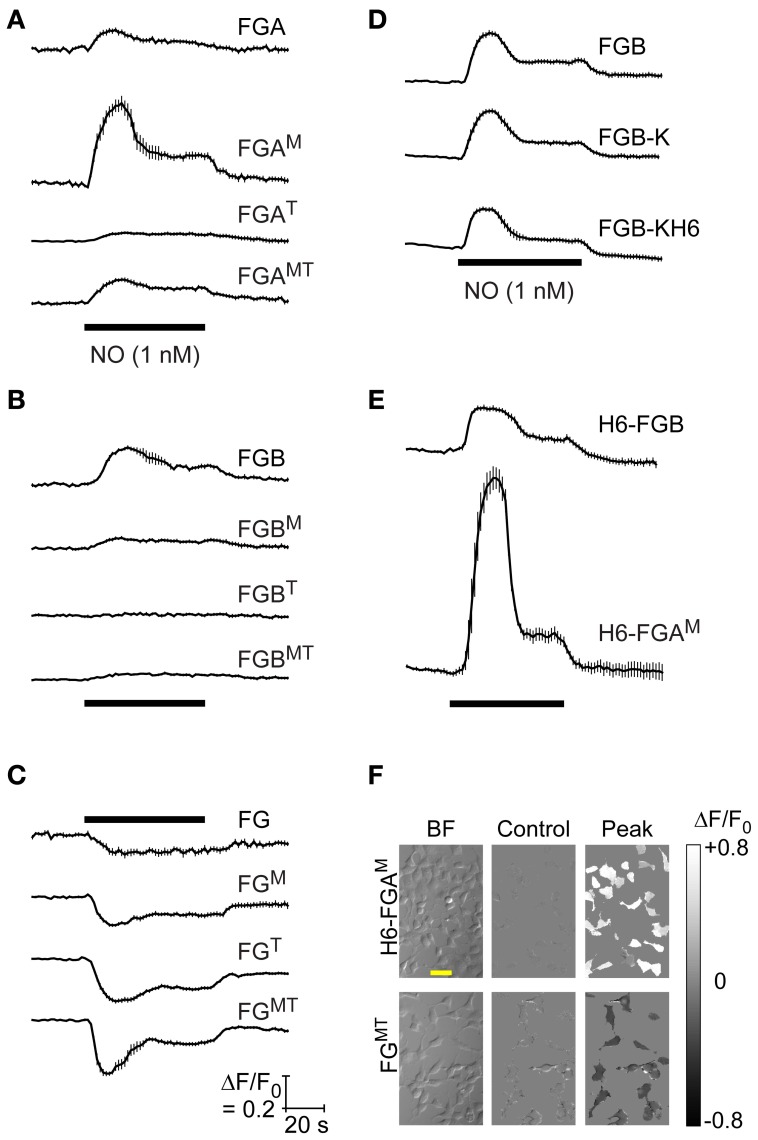
**Responsiveness to NO of HEK_GC/PDE5_ cells transfected with FlincG variants. (A–E)** Representative recordings (from 3 to 4 experiments) of HEK_GC/PDE5_ cells transfected with the indicated FlincG variants (grouped as in Figure 1) and exposed to 1 nM NO for 1 min (horizontal bars). Traces are averages of 5–20 cells; vertical bars are ± SEM. Calibrations (**C**, bottom) apply to all panels. **(F)** Sample brightfield images (“BF”, left), together with the baseline fluorescence (“Control”, center) and maximal NO-induced change in fluorescence (“Peak”, right), coded into the grayscale shown on the extreme right, of cells transfected with H6-FGA^M^ (top) and FG^MT^ (bottom); scale bar (in the top left image) = 50 μm.

To test how the effects of these molecular variations would translate in a more physiological setting, transfected HEK_GC/PDE5_ cells were superfused (1 min) with NO to stimulate endogenous cGMP generation and the responses recorded using fluorescence imaging. The NO concentration chosen (1 nM) was one that gives a maximal response when the cells are infected with adenoviral FlincG (Batchelor et al., 2010; Wood et al., 2011). In general, the pattern of responses of the FlincG variants to NO recorded at the single-cell level (Figure 2) was similar to that observed by FACS analysis. Responses to NO were triphasic, being composed of a transient peak followed by a prolonged plateau. This response shape reflects a rapid stimulation of NO-activated guanylyl cyclase, raising cGMP, and a slow increase in activity of the cGMP-stimulated phosphodiesterase-5, gradually enhancing the rate of cGMP hydrolysis (Mullershausen et al., 2004; Halvey et al., 2009; Batchelor et al., 2010). Within the A-tail series, FGA^M^ gave the largest response to NO whereas the variant having the published FlincG domains (FGA) gave only a low-amplitude fluorescence change (Figure 2A). In the B-tail series, FGB was the best sensor (Figure 2B) and the tailless FG group all gave inverted responses that were largest with FG^T^ and FG^MT^ (Figures 2C,F).

From these results, two of the variants were selected for protein purification and spectroscopic analysis, and possible crystal structure determination: FGB, because it was most like the adenoviral FlincG and its response in the HEK_GC/PDE5_ cells resembled that of the adenoviral construct; and FGA^M^, because of its superior response amplitude (despite low basal fluorescence). Enzyme-cleavable hexahistidine tags were added to facilitate protein purification. The C-terminal tag requires a preceding lysine residue to render it carboxypeptidase-sensitive and this residue would remain in the protein following tag removal. As judged by FACS analysis, adding a lysine to FGB (FGB-K) increased the median basal fluorescence in HEK_GC/PDE5_ cells by about 2-fold, an effect that persisted with the further addition of a hexahistidine tag (FGB-KH6); responses to 8-bromo-cGMP were not obviously changed by either modification (Figure 1E). Cellular NO-sensitivity recorded at the single cell level was also unaltered (Figure 2D). N-terminal tags were supplied by the pTriEx-4 vector. HEK_GC/PDE5_ cells transfected with H6-FGB displayed no major changes in basal fluorescence or in responsiveness to 8-bromo-cGMP (Figure 1F) and NO (Figure 2E). Unexpectedly, adding a N-terminal tag to FGA^M^ had a marked effect. In FACS spectra, the median basal intensity of cells increased almost 7-fold compared with the untagged version (see Figure 1C) to become one of the brightest, rather than dimmest, of the variants. Furthermore, the mean shift with 8-bromo-cGMP was the largest, amounting to about twice the increase recorded using FGB (Figures 1C,F). The results of the FACS screen were verified by single-cell imaging, which showed H6-FGA^M^ to produce the largest response to superfusion of 1 nM NO (Figures 2E,F).

### Characterization of purified H6-FGB and H6-FGA^M^ proteins

**Figure 3:**
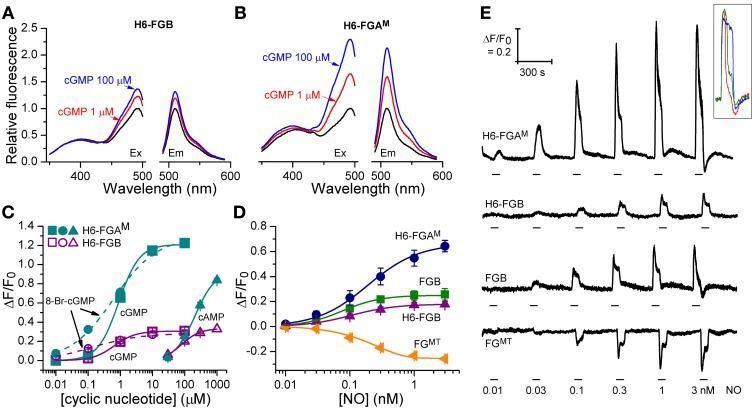
**Cyclic nucleotide sensitivity of H6-FGA^M^ and H6-FGB proteins and NO-sensitivity of HEK_GC/PDE5_ cells expressing selected FlincG variants. (A,B)** Sample excitation (left) and emission (right) spectra of H6-FGB **(A)** and H6-FGA^M^ **(B)** proteins in the absence (black) and presence of 1 μM (red) and 100 μM (blue) cGMP, normalized to the peaks in the absence of cGMP. **(C)** Cyclic nucleotide concentration-response curves for the two proteins. Points are means of three determinations ± SEM. **(D)** NO concentration-response curves in HEK_GC/PDE5_ cells transfected with the indicated FlincG variants (*n* = 3–5). **(E)** Representative recordings of responses to NO contributing to panel **D**. NO concentrations (specified at bottom) were superfused for the time indicated by the horizontal bars. Traces are means of 11–24 cells; the calibration (top left) applies to all of them. The inset illustrates the heterogeneity in the response profile amongst cells in a given experiment often (but not always) observed at the higher NO concentrations. Also at the higher concentrations, responses were sometimes followed by small, rapid undershoots and small, slow secondary fluorescence increases (or decreases in the case of FG^MT^, bottom trace), as was observed previously with adenoviral FlincG-infected HEK_GC/PDE5_ cells (Batchelor et al., 2010).

The fluorescence spectra of H6-FGB and H6-FGA^M^ proteins were similar, showing prominent excitation peaks at 410 and 480 nm, and emission peaks at 510 nm (Figures 3A,B), comparable to the spectrum reported for the original FlincG (Nausch et al., 2008). On addition of cGMP, only the major excitation and emission peaks at 480 nm and 510 nm, respectively, substantially increased in amplitude, the maximum change with H6-FGA^M^ being 4-fold larger than with H6-FGB. Concentration-response curves (Figure 3C) showed that H6-FGB and H6-FGA^M^ proteins were respectively 120- and 230-fold selective for cGMP over cAMP (Table 1). The half-maximally effective concentrations (EC_50_ values) of cGMP were in the same range for the two biosensors: 0.89 μM for H6-FGA^M^ and 0.64 μM for H6-FGB, with the Hill slopes (1.3–1.4) meeting expectations should the protein have two cGMP-binding sites, both of which need to be occupied for the conformational change and associated fluorescence increase to occur (Batchelor et al., 2010). It was also of interest to determine the potency of 8-bromo-cGMP because this derivative is used to generate a fluorescent signal in the FACS experiments (see above) and for testing cGMP biosensors expressed in other cell types (see below). With both H6-proteins, 8-bromo-cGMP was more potent than cGMP but the concentration-response curves were shallow, with Hill slopes of about 0.6 (Figure 3C; Table 1).

**Table 1:** **Concentration-response curve parameters for purified FlincG proteins**.

**FlincG protein**	**EC_50_ (μM)**	***n_H_***	**R_max_**
**H6-FGA^M^**			
cGMP	0.89 ± 0.05	1.36 ± 0.16	1.21 ± 0.02
8-Br-cGMP	0.62 ± 0.23	0.64 ± 0.17	1.29 ± 0.08
cAMP	204 ± 25	1.43 ± 0.18	0.92 ± 0.05
**H6-FGB**			
cGMP	0.64 ± 0.004	1.32 ± 0.01	0.31 ± 0.001
8-Br-cGMP	0.15 ± 0.09	0.60 ± 0.17	0.29 ± 0.01
cAMP	75.4 ± 4.0	1.48 ± 0.08	0.33 ± 0.01

n_H_ is the Hill slope and R_max_ the maximum response (ΔF/F_0_); n = 3.

### Evaluation of selected FlincGs as cellular cGMP biosensors

From the results obtained above, the properties of four potential biosensors were scrutinized using the model HEK_GC/PDE5_ cells. Those selected for investigation were the two H6-tagged proteins (H6-FGA^M^ and H6-FGB), FGB, and FG^MT^, the latter being the tailless variant that dimmed the most in response to cGMP (and 8-bromo-cGMP). Firstly, concentration-response relationships following superfusion (1 min) of NO in clamped concentrations were determined. All the biosensors responded in an NO concentration-dependent manner with the responses peaking at about 1 nM NO (Figures 3D,E). Moreover, they all generated similar triphasic response shapes (see above) at the higher NO concentrations, suggesting sensitivity of the biosensors to endogenous cGMP in the 0.1–10 μM range (Batchelor et al., 2010). Particularly at the highest concentration (3 nM NO), there was some cell-to-cell variation in the shape of the response although the rising phases were closely similar (Figure 3E inset), indicating that the heterogeneity reflects variations in phosphodiesterase-5 (rather than guanylyl cyclase) activity (Batchelor et al., 2010). On re-inspection of the individual cell recordings, a similar variation was found in the results of an earlier study when these cells were infected with adenoviral FlincG (Batchelor et al., 2010). The potency of NO was highest with FGB and H6-FGB (EC_50_ = 76 and 104 pM), falling 2- to 3-fold lower with H6-FGA^M^ and FG^MT^ (EC_50_ = 190 pM) but the Hill slopes were all similar (Figures 3D,E; Table 2). The most obvious difference was in the response amplitude, which was in the order H6-FGA^M^ > >FGB = FG^MT^ > H6-FGB. After averaging, H6-FGA^M^-transfected cells detected 10 pM NO in 3 out of 5 experiments whereas the other biosensors responded to this concentration once (in 3 or 4 experiments). All the biosensors responded to 30 pM NO in each experiment.

**Table 2:** **NO concentration-response curve parameters in FlincG-transfected HEK_GC/PDE5_ cells**.

**FlincG**	**EC_50_ (pM)**	***n_H_***	***R*_max_**
H6-FGA^M^	191 ± 37	1.10 ± 0.09	0.66 ± 0.03
H6-FGB	104 ± 25	1.26 ± 0.19	0.18 ± 0.01
FGB	76 ± 12	1.45 ± 0.18	0.25 ± 0.02
FG^MT^	190 ± 14	1.36 ± 0.09	−0.26 ± 0.005

n_H_ is the Hill slope and R_max_ the maximum response (Δ F/F_0_); n = 3–5.

**Figure 4:**
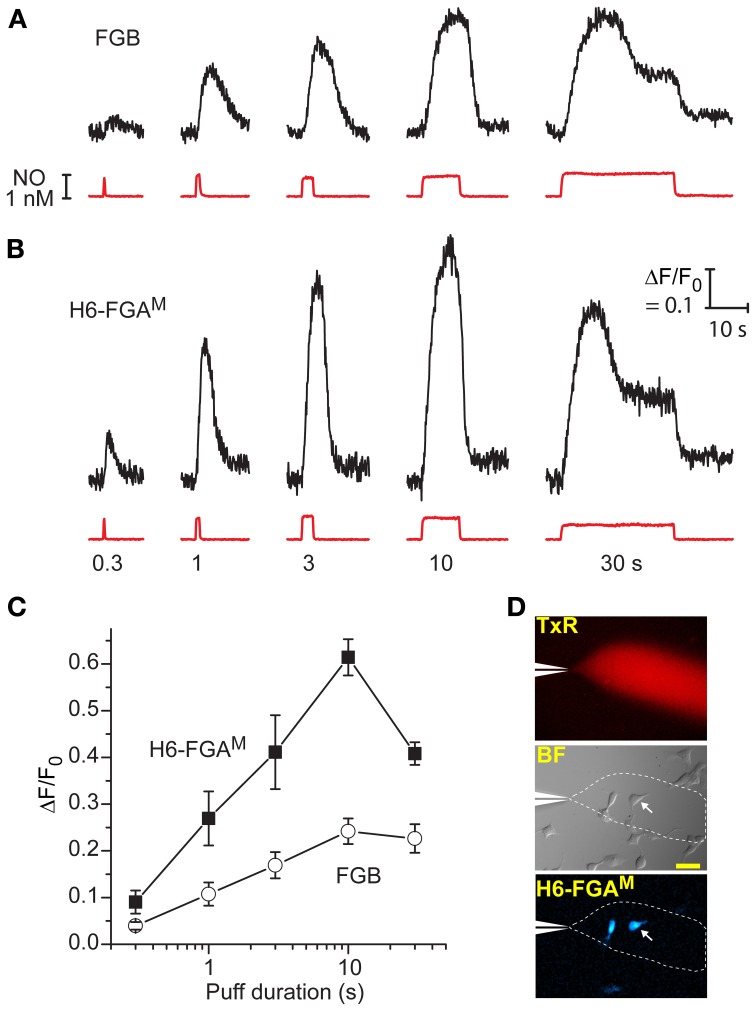
**Comparison between H6-FGA^M^ and FGB as sensors of NO puffs when expressed in HEK_GC/PDE5_ cells. (A,B)** Representative traces of fluorescence changes to NO puffs of different durations (black traces); the NO applications are quantified from the Texas Red dye intensity (red traces), with the applied durations specified underneath. There was a 5-min interval between applications and each result is from a single cell. Calibration (in **B**) applies to both sets of data. **(C)** Mean responses (± SEM) to the different puff durations in four (H6-FGA^M^) or seven (FGB) cells in three experiments. **(D)** Images showing the spread of Texas Red from the puffer pipette (top) within a field of cells (brightfield, middle), two of which are fluorescent (bottom); the arrowed cell is the source of the traces in **(B)**; scale bar (middle) = 50 μm.

A second series of tests aimed to probe the dynamic responsiveness of the two leading cGMP biosensors, FGB and H6-FGA^M^, by rapidly administering puffs of NO of varying duration to transfected cells from a nearby pipette (see Figure 4D). With 1 nM NO in the pipette, puffs lasting 300 ms produced detectable responses through both biosensors, with H6-FGA^M^ again giving the higher amplitude (Figures 4A,B). The responses increased in size with longer puffs, reaching a peak with 10-s applications, when the fluorescent increase with H6-FGA^M^ was almost 3-fold higher than with FGB. Half-maximum responses corresponded to a puff duration of about 1.3 s for both biosensors (Figure 4C). With H6-FGA^M^, the peak response with a long (30-s) puff was lower than with a shorter (10-s) one, whereas the amplitudes of the two were the same with FGB. This result is expected from H6-FGA^M^ having a lower cGMP affinity than FGB (Table 1): the sequence of 1 nM NO applications leads to a progressive build-up of activated phosphodiesterase-5 which results in lower cGMP concentrations during the 30-s application than during the preceding 10-s application; a lower-affinity biosensor would register this fall whereas the higher-affinity biosensor would remain saturated (see Figure 5 of Batchelor et al., 2010). In all cases, the timing of the response onset and start of recovery coincided with the arrival and disappearance of NO, after allowing for its diffusion through unstirred layers surrounding the cells (Batchelor et al., 2010), indicating that the kinetic properties of the two biosensors are immeasurably fast at these subsecond time-scales.

### Sensitivity to pH

**Figure 5:**
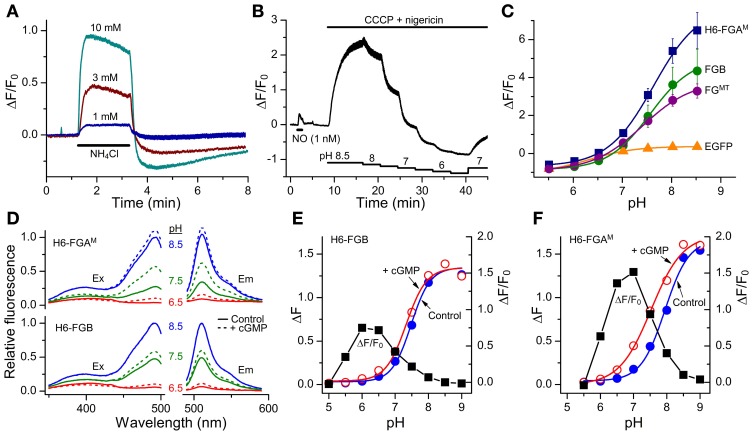
**Sensitivity of FlincG variants to pH. (A)** Responses of HEK_GC/PDE5_ cells expressing FGB to 2-min applications of NH_4_Cl (horizontal bar). Traces are means from 40 cells; vertical bars are ± SEM. **(B)** HEK_GC/PDE5_ cells expressing FGB were initially stimulated with NO (1 nM; 1 min), after which the combination of CCCP (20 μM) and nigericin (10 μM), which results in an equalization of intracellular and extracellular pH, was superfused at different pH values (as indicated by the stepped horizontal bars below). The trace is the average of 10 cells; vertical bars are ± SEM. **(C)** Summary pH titration data obtained as in panel **(B)** on HEK_GC/PDE5_ cells expressing different FlincGs or EGFP (*n* = 3). **(D)** Sample excitation (Ex) and emission (Em) spectra of purified H6-FGA^M^ and H6-FGB proteins in the absence (solid lines) or presence (dashed lines) of 100 μM cGMP, at pH 6.5 (red), 7.5 (green) and 8.5 (blue), normalized to the peak at pH 8.5 in the absence of cGMP. **(E,F)** pH-titration data for the fluorescence (arbitrary units) of the purified FlincG proteins in the absence and presence of 100 μM cGMP (left-hand ordinate), and the dynamic ranges, expressed as Δ*F*/*F*_0_, at each pH (black squares; right-hand ordinate).

The classical intracellular pH modifier, NH_4_Cl (1–10 mM), was perfused onto HEK_GC/PDE5_ cells transfected with FGB. An increase in fluorescence occurred that waned gradually during the exposure (Figure 5A). On washout, the fluorescence fell below the resting level and then recovered slowly. The shapes of these responses closely resemble the usual NH_4_Cl -induced alkalinization and after-acidification of intracellular pH (Roos and Boron, 1981). For a more direct test, the cells were treated with a mixture of ionophores (nigericin plus CCCP) that result in the equalization of intracellular and extracellular pH (Kneen et al., 1998). In a sample experiment (Figure 5B) titration of the pH between 8.5 and 5.5 led to marked changes in FGB fluorescence that were up to 10-fold larger than that generated previously (via cGMP) by a maximally effective concentration of NO and that were half-maximal at around pH 7.5. The other FlincG variants tested in this way, namely H6-FGA^M^ and FG^MT^ (which dims in response to cGMP) showed similar pH sensitivity (Figure 5C), with apparent pK_a_ values of about 7.5 and Δ *F*/*F*_0_ dynamic ranges of 4.7–8.5 (Table 3). The sensitivity to alkaline shifts was much higher than that of EGFP (Δ*F*/*F*_0_ = 0.36 at pH 8.5; Figure 5C). From the calibrations, the peak responses to NH_4_Cl in intact FGB-transfected HEK_GC/PDE5_ cells (Figure 5A) correspond approximately to increases in intracellular pH of 0.04 units (1 mM NH_4_Cl), 0.21 units (3 mM) and 0.4 units (10 mM). These changes are consistent with 20 mM ammonia raising the pH of HEK293T cells by about 1 unit (Schuhmann et al., 1997; Lang et al., 2003).

**Table 3:** **pH-sensitivity parameters in FlincG-transfected HEK_GC/PDE5_ cells**.

**FlincG**	**pK^′^_*a*_**	***n_H_***	**A**	**B**
FGA^M^	7.63 ± 0.05	0.93 ± 0.04	−0.68 ± 0.02	8.45 ± 0.43
FGB	7.56 ± 0.03	1.01 ± 0.02	−0.86 ± 0.005	5.88 ± 0.18
FG^MT^	7.41 ± 0.02	0.86 ± 0.02	−0.91 ± 0.008	4.73 ± 0.09
EGFP	6.33 ± 0.03	0.94 ± 0.04	−1.01 ± 0.03	1.38 ± 0.04

pK_a_' is the apparent pK_a_, n_H_, the Hill slope; A and B are constants in the titration equation given in material and methods and correspond to the baseline of the curve (A) and the ΔF/F_0_ dynamic range (B); n = 3.

The pH sensitivity of purified H6-FGA^M^ and H6-FGB proteins was assessed with and without a saturating concentration of cGMP (100 μM) using spectrofluorometry. With both proteins, the excitation peak at 480 nm was highly pH-sensitive, as was the emission peak at 510 nm (Figure 5D). Titration curves showed apparent pK_a_ values of 7.9 and 7.5 for H6-FGA^M^ and H6-FGB, respectively, in the absence of cGMP (Figures 5E,F; Table 4). In the presence of cGMP, both curves shifted in the acidic direction, the shift with H6-FGA^M^ being larger than with H6-FGB. The net result was that the pH giving the maximal cGMP-induced change in fluorescence (Δ *F*/*F*_0_) was lower for H6-FGB (pH 6.0) than for H6-FGA^M^ (pH 7.0). The larger response of H6-FGA^M^ to cGMP seen previously at pH 7.5 (Figure 3C; see also Figure 3E for cell data) also applied at the pH optima, where the peak Δ*F*/*F*_0_ of H6-FGA^M^ was twice that of H6-FGB (Figures 5E,F).

**Table 4:** **pH-sensitivity of purified FlincG proteins**.

**FlincG protein**	**pK^′^_*a*_**	***n_H_***
**H6-FGA^M^**		
Control	7.94 ± 0.07	1.20 ± 0.20
cGMP (100 μM)	7.53 ± 0.08	0.91 ± 0.15
**H6-FGB**		
Control	7.48 ± 0.05	1.51 ± 0.24
cGMP (100 μM)	7.33 ± 0.06	1.41 ± 0.25

pK_a_' is the apparent pK_a_ and n_H_ the Hill slope.

### Transfection of neural cells

**Figure 6:**
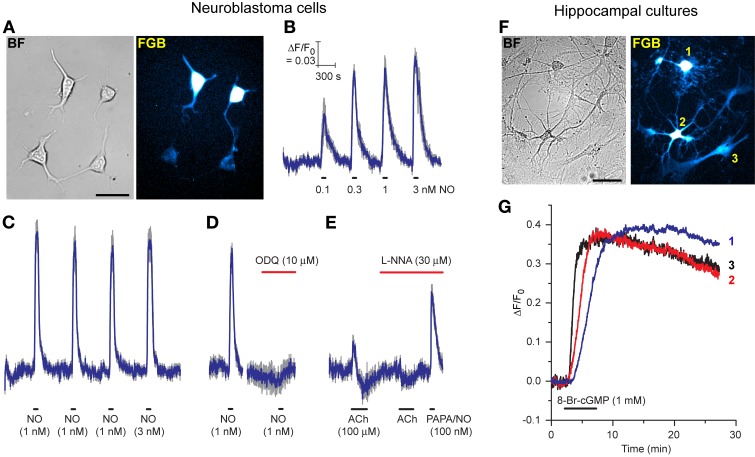
**Expression of FGB in differentiated N1E-115 neuroblastoma cells (A–E) and rat hippocampal cell cultures (F,G). (A)** Brightfield (left) and fluorescent (right) images of neuroblastoma cells (two transfected, two untransfected); scale bar = 50 μm. **(B)** Response to increasing clamped NO concentrations applied at the horizontal bars (5 cells). **(C)** Response to three consecutive 1 nM NO applications followed by 3 nM NO (10 cells). **(D)** Inhibition of the response to 1 nM NO by ODQ (8 cells). **(E)** Response to acetylcholine (ACh) in the absence and presence of L-nitroarginine (L-NNA) with application of a high concentration of PAPA/NO at the end (4 cells). Blue lines in B-E are means; gray lines = SEM. **(F)** Brightfield (left) and fluorescent (right) images of a rat hippocampal cell culture. Cells numbered 1, 2, and 3 are a putative oligodendrocyte, neurone and astrocyte, respectively, based on their morphology. Scale bar = 50 μm. **(G)** Responses of these 3 cells to superfusion of 8-bromo-cGMP.

Considering the disappointing transfection efficiency observed at the outset with the AdV-FlincG DNA plasmid, tests were carried out to determine if the superior properties of the FlincG variants in HEK_GC/PDE5_ cells translated into greater versatility of transfection, particularly for neurones which pose particular difficulties in this regard. As a first step, we attempted to transfect differentiated N1E-115 neuroblastoma cells with FGB. These cells have long been known to generate cGMP on stimulation of cholinergic and other receptors (Matsuzawa and Nirenberg, 1975; Saito and Deguchi, 1979), effects now known to be mediated through endogenous NO formation (Hu and El-Fakahany, 1993). The transfection was routinely successful with around 25% of cells showing strongly fluorescent cell bodies and more weakly fluorescent neurites (Figure 6A). On superfusion of NO in clamped concentrations (0.1–3 nM), the fluorescence increased and then recovered back to baseline on washout; the NO EC_50_ was approximately 100 pM (Figure 6B). Responses to 1 nM NO were repeatable (Figure 6C) and were abolished by the inhibitor of NO-activated guanylyl cyclase ODQ (Figure 6D), confirming mediation by cGMP. On superfusion of acetylcholine to elicit endogenous NO synthesis, the fluorescence increased but then faded during the application (after about 1 min) to fall below baseline, before recovering on washout of acetylcholine (Figure 6E). In the presence of the NO synthase inhibitor, L-nitroarginine (30 μM), the increase in fluorescence was abolished, but a small undershoot remained. The undershoots seen with and without L-nitroarginine possibly reflect a transient cell acidification being registered by the biosensor (Seo et al., 1994; Wood et al., 2011). Superfusion of a high concentration of the NO donor PAPA/NO (100 nM) at the end of the experiment (Figure 6E) indicated that the peak acetylcholine-evoked increase in fluorescence corresponded to about half the maximum amplitude or, from Figure 6B, about 100 pM NO.

In the light of these positive results with neuroblastoma cells, pilot experiments were carried out on primary cultures of rat hippocampal and dorsal root ganglion (DRG) cells. Despite minimal attempts at optimization, cells of different types in mature hippocampal cultures were successfully transfected with FGB: Figure 6F shows a putative oligodendrocyte, neurone and astrocyte fluorescing in a single field of cells. On superfusion of 8-bromo-cGMP these cells all responded, signifying that they expressed a functional cGMP biosensor.

**Figure 7:**
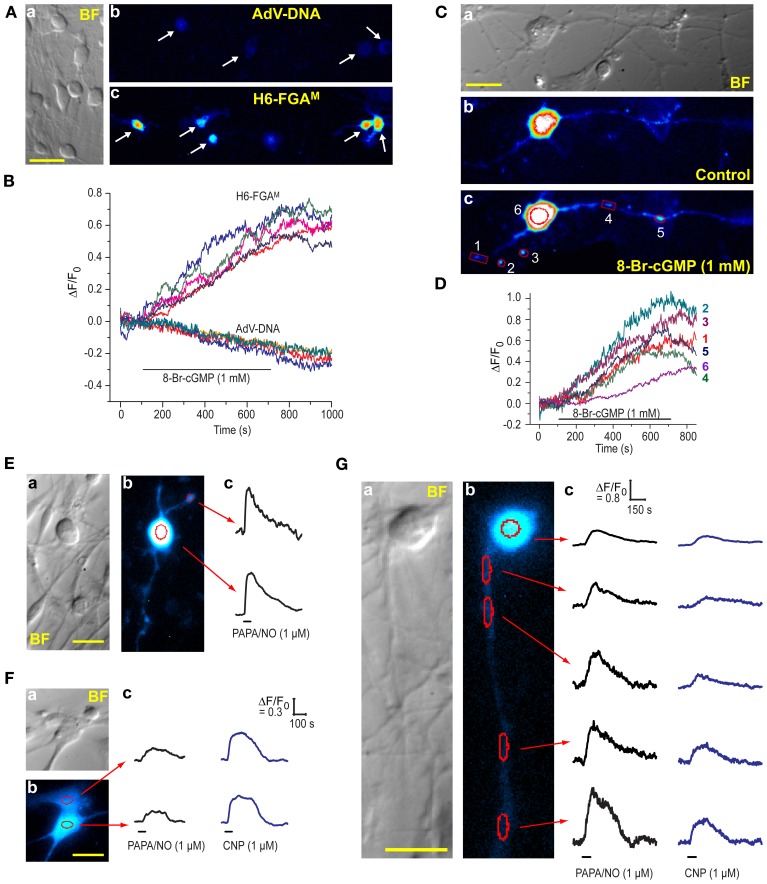
**Expression of H6-FGA^M^ in DRG cultures. (A)** (a) Brightfield image of a typical culture showing neurones of different sizes and an underlay of flatter, glial-like cells. (b,c) Fluorescence images of cultures after attempted transfection with AdV-FlincG plasmid DNA (b) and H6-FGA^M^ (c) and exposure to 1 mM 8-bromo-cGMP for 10 min. Scale bar (a) = 50 μm. **(B)** Time-courses of fluorescent changes in the individual cells arrowed in **(A**b**)** and **(A**c**)** during superfusion of 8-bromo-cGMP. **(C)** High-power brightfield (a) and fluorescence (b,c) images of a transfected neurone before (b) and after (c) superfusion of 8-bromo-cGMP (1 mM) for 8 min. The images in (b) and (c) were processed identically. Scale bar (a) = 25 μm. **(D)** Time-course of the changes in fluorescence in the number-coded puncta and cell soma demarcated in red in (c). Relatively large regions of interest were used for the puncta to accommodate their movement during the experiment. This procedure should not have affected the signal amplitude but may have increased the noise. **(E–G)** Neuronal **(E,G)** and glial-like **(F)** cells transfected with H6-FGA^M^ and exposed to PAPA/NO **(E)** or PAPA/NO followed 20–30 min later by CNP **(F,G)**. Each panel **(E–G)** shows pairs of brightfield (a) and fluorescent (b) images; scale bars = 25 μm. Traces (c) are from the regions outlined in red in (b); black horizontal bars = periods of agonist application; in **(E)** only one punctum (at the top) was analyzed because the others, despite obviously responding, were too motile; calibrations in **(F**c**)** also apply to the traces in **(E**c**)**.

The DRG cultures comprised varying sized neurones having prominent rounded cell bodies accompanied by a background of glia (satellite cells and Schwann cells) and fibroblasts (Figure 7A), as is normally found in these cultures (Fields et al., 1978). We attempted to transfect the cells with H6-FGA^M^ and, to check results with HEK_GC/PDE5_ cells (Figure 1B), also with AdV-FlincG plasmid DNA. No fluorescence above background was detected with AdV-FlincG plasmid DNA and, on superfusion of 8-bromo-cGMP, only a continuing bleaching of the autofluorescence was recorded (Figures 7A,B), implying that the cells lacked a functional biosensor. With H6-FGA^M^, on the other hand, neurones (and non-neuronal cells; see below) were clearly transfected (Figure 7Ac) and they responded to 8-bromo-cGMP with a slow increase in fluorescence (Figure 7B), ultimately reaching amplitudes comparable to those seen with this biosensor in HEK_GC/PDE5_ cells exposed to maximal NO stimulation (e.g., Figure 3D). With higher-power imaging, neuronal processes could be discerned (Figure 7C), although their baseline fluorescence was relatively low, reflecting their thinness. On perfusion of 8-bromo-cGMP, the fluorescence of the processes increased along their visible lengths (see below). Varicosities, approximately 1–2 μm in diameter, responded more obviously and with a fluorescence change of similar amplitude to, but faster than, that seen in the soma, (Figure 7D).

NO and a member of the natriuretic peptide family, namely C-type natriuretic peptide (CNP), are both important signaling molecules for DRG neurones, particularly during their development: NO provides neuroprotection by elevating cGMP in Schwann cells, resulting in the release of neurotrophic factors (Thippeswamy et al., 2005) whereas CNP contributes to the growth of axons and their connectivity within the spinal cord (Schmidt et al., 2007; Kishimoto et al., 2008; Zhao and Ma, 2009). CNP acts through natriuretic peptide receptor-2, a membrane-spanning guanylyl cyclase-coupled receptor expressed in DRG neurones (Schmidt et al., 2007; Kishimoto et al., 2008). To test the ability of H6-FGA^M^ to register agonist-induced cGMP elevations in DRG cells, saturating concentrations of PAPA/NO (1 μM) or CNP (1 μM) were superfused (1 min). Responses were elicited from almost every transfected cell examined (9/11 and 12/12 cells, respectively; 8 coverslips, 2 separate platings), whether they were neuronal (Figure 7E) or non-neuronal in appearance (Figure 7F). Responsiveness of both cell populations to NO in DRG cultures agrees with a previous report using cGMP immunocytochemistry (Thippeswamy and Morris, 2001). In the 15 cells where both agonists were applied 20–30 min apart, the relative response amplitudes differed in an unsystematic way (Figures 7F,G) but, in all instances, the decay was slow, taking 3–5 min after agonist washout for the baseline to be resumed. A neurone having a long (>100 μm) neurite that remained physically stable during the recording period was fortuitously captured in one field (Figure 7G). Responses to both PAPA/NO and CNP were seen in the soma and along the length of the neurite, and were consistently larger with PAPA/NO than with CNP. The amplitudes increased progressively with distance from the soma (reaching a 1.5-fold change with PAPA/NO), a finding that may partially be related to a progressively diminishing basal fluorescence, which became close to background at the most distal point. The results indicate that, at least at high concentration, NO and CNP may have multiple cellular targets in the DRG, all of which appear to exhibit low cGMP phosphodiesterase activity, thereby allowing global build-up of cGMP irrespective of the presumably different subcellular locations (cytosolic vs. membrane-associated) of its synthetic enzymes.

## Discussion

In an attempt to generate a useful cDNA plasmid for cGMP biosensing in a variety of cell types, it transpired that the adenoviral FlincG, which has allowed successful recordings of cGMP signals in smooth muscle, HEK293T cells and cardiac fibroblasts (Nausch et al., 2008; Batchelor et al., 2010; Miller et al., 2011; Wood et al., 2011), and a plasmid FlincG, both kindly provided to us by the originator laboratory, coded for a sequence that differed from the one that they originally reported (Nausch et al., 2008). In evaluating the importance of the differences, restoration of an arginine (from cysteine) in the cpEGFP region was of clear benefit for expressing a functional biosensor in HEK293T cells using a cDNA plasmid. When driven by the adenoviral vector, a higher protein expression may compensate for this disadvantageous mutation. It is unclear how the mutation arose but, according to the distributer's website (Addgene: http://www.addgene.org/), it is present in DNA sequences for all the original FlincG variants (α-, β- and δ-FlincG).

The other region of concern was in the 17-amino acid C-terminal tail which, apart from the first 3 amino acids, was different from the stated sequence. Again, this divergent tail appears in all the sequences deposited in the distributor's website. The change from the bovine PKG sequence could be explained by two point mutations: deletion of a cytosine in the fourth codon (altering the reading frame and creating a premature stop codon) together with a mutation in the triplet immediately preceding this stop codon. The authors state that extending this tail to include the PKG catalytic domain or complete removal of the tail had no effect on overall fluorescent intensity changes (Nausch et al., 2008). In contrast, we find the tail region to have a major effect on cGMP biosensing, such that restoring the “correct” PKG sequence (in the absence of further modification) gave only poor responsiveness to cGMP (or 8-bromo-cGMP), as did its complete removal. Thus, on this evidence, the “incorrect” tail (tail B) appears to be superior. By analogy with inferences made for GCaMP3 (Tian et al., 2009), one explanation might be that tail B codes for a nuclear exclusion sequence [L(X)_2−3_L(X)_2−3_L; (Bogerd et al., 1996; la Cour et al., 2004)] but, as variants lacking a tail (i.e., FG^T^ and FG^MT^) and one variant with the “correct” tail (H6-FGA^M^) functioned at least as well as FGB, this explanation appears inadequate.

In addition to the importance of the tail region, a second discrepancy between our results and the original report on FlincG (Nausch et al., 2008) concerns pH sensitivity. The authors of this report claim that FlincG is insensitive to pH in the physiological range because of its low pK_a_ (6.1) but this conclusion is drawn from a plot of the cGMP-induced changes in fluorescence at different pH values (their Supporting Information Figure 7), so that information on how basal fluorescence varies with pH is lost. Our results both with transfected cells and purified proteins show that basal fluorescence is highly pH-dependent, giving apparent pK_a_ values of about 7.5. In this respect, our data are in good agreement with expectations from the properties of cpEGFP, whose apparent pK_a_ is reported as 7.7 (Akemann et al., 2001) and 7.4 (Wang et al., 2008). Indeed, the more physiological pH optimum displayed by H6-FGA^M^ for the cGMP-induced fluorescence increase (compared with H6-FGB; Figures 5E,F) helps explain its superior dynamic range when expressed in cells. For example, assuming HEK293T cells have an intracellular pH of 7.5 (Willoughby et al., 2005), the maximum cGMP-induced fluorescence change (Δ *F*/*F*_0_) for H6-FGB and H6-FGA^M^ would be 0.22 and 0.94, respectively, based on protein data while the maximum changes in NO-stimulated HEK_GC/PDE5_ cells amounted to 0.18 and 0.66, respectively. The values are in good agreement (both about 4-fold different) bearing in mind that the cell responses are expected to be of lower amplitude because they were measured with broader band-pass filter sets compared with the monochromator-controlled slit-width used for the purified proteins. At the lower pH values of 7 and 6.5, the relative maximum fluorescence changes of the two sensors should differ less, by 3-fold and 2-fold, respectively (from Figures 5E,F).

Clearly, just as with other cpEGFP-based biosensors, such as GCaMPs (Nakai et al., 2001; Wang et al., 2008; Zhao et al., 2011), it is important to be mindful of pH-induced artifacts in live cell imaging. As a case in point, using FGB-transfected HEK_GC/PDE5_ cells as detectors for NO released from NMDA-stimulated brain slices, we observed fluorescent undershoots that were NO-independent and attributed to transient acidifications brought about by products of metabolic stimulation, notably lactate (Wood et al., 2011). The small NO-independent undershoots observed in neuroblastoma cells exposed to acetylcholine (Figure 6E) might have a similar origin.

We also examined the effect of M- and T-mutations that contributed to the engineering of GCaMP3 from GCaMP2 (Tian et al., 2009), and the interaction of these mutations with the type of tail. In the structure of GCaMP2, the T-residue (T116) faces the inside of the β-barrel of cpEGFP and thus directly influences the protonation state of the chromophore (Wang et al., 2008). Mutating this residue to valine increases excited-state proton transfer in EGFP (Kummer et al., 2000) and enhances the fluorescent response of GCaMP2 to Ca^2+^ by 2-fold (Akerboom et al., 2009). On its own, the T-mutation in FlincG variants had a deleterious effect on the responsiveness to cGMP with both tail types, but enhanced the cGMP-induced dimming of the tailless variant (FG). The M-residue faces outside the β-barrel and mutating it to a hydrophilic residue was originally one of the cycle-3 GFP mutations that improved brightness and reduced aggregation (Crameri et al., 1996). One of the other two cycle-3 mutations (V164A) is already retained in the cpEGFP region of GCaMP2/3 and FlincG. Again, the M-mutation in FlincG variants offered little obvious improvement, with the exception of the variant with the “correct” tail A (i.e., FGA^M^) which gave enhanced cGMP response amplitudes. With double mutations, the only notable benefit was in the amplitude of dimming of the tailless variant (FG^MT^). Altogether, our results point to a lack of generalizability of the effect of mutations across FlincG variants, and an inability to extrapolate findings with GCaMPs to other cpEGFP-based biosensors, although in specific cases one or both mutations can lead to improvements. Obviously, structural information is needed to interpret the effects of the mutations mechanistically but, unfortunately, initial attempts at obtaining protein crystals (with H6-FGA^M^) were not successful.

Finally with respect to the development of FlincG-based biosensors, the attachment of an N-terminal tag (comprising a hexahistidine tag, Protein S-tag and an enterokinase cleavage site) led to an unexpected improvement in one of the variants (FGA^M^). Importantly, the tag seemed solely to enhance basal fluorescence (which was unusually low without the tag), without compromising functional responsiveness. A beneficial effect of an N-terminal tag, in this case supplied by the vector pRSET, has also been reported for GCaMP2, its effect being attributed to an enhanced thermal stability at 37°C (Tallini et al., 2006). Although the tag we used is different from the pRSET version (but both plasmids supply a hexahistidine tag), our observations are consistent with such an interpretation and, while the variant is named H6-FGA^M^, it is not necessarily the hexahistidine tag that confers the advantage.

In evaluating the potential utility of the improved FlincG variants, the main criteria are high sensitivity and selectivity for cGMP, high basal fluorescence, high signal-to-noise ratio, and rapid kinetics. In terms of the latter, there was no evidence that the kinetics of any of the biosensors was rate-determining, although the most rigorous testing (brief puff applications of NO) was carried out only on the two leading variants. Hence, the modified biosensors are likely to operate at least as rapidly as the original FlincG (Nausch et al., 2008; Batchelor et al., 2010). Based on all the above criteria, two of the variants (FGB and H6-FGA^M^) showed particular promise in the model HEK293T cells. A third variant, FG^MT^, is also potentially interesting in that it shows high basal fluorescence that dims in the presence of cGMP but the maximum response amplitude was no better than with FGB. While we have not determined the potency of cGMP on FGB directly, the EC_50_ for NO (76 pM) in HEK_GC/PDE5_ cells was essentially identical to the value obtained using the original FlincG in the same cells (70 pM; Batchelor et al., 2010; Wood et al., 2011), implying a similar affinity for cGMP (EC_50_ = 0.17 μM; Nausch et al., 2008), whereas the EC_50_ for cGMP on H6-FGA^M^ was 5-fold lower at 0.89 μM (Table 1). Consistent with cGMP also having a lower potency for H6-FGA^M^ in cells, the EC_50_ for NO in HEK_GC/PDE5_ cells was shifted upwards to 190 pM and, unlike FGB, the biosensor registered the fall in cGMP response amplitude taking place at the end of the NO puff-application sequence (Figure 4B). Assuming the associated changes in cGMP are physiological, the lower affinity biosensor, which saturates at about 10 μM cGMP, may be preferable to the original FlincG, which saturates at about 2 μM cGMP. Moreover, because of the larger change in fluorescence of H6-FGA^M^, the detectability of small changes in cGMP is minimally compromised, as was evident with the subsecond puff applications of NO (Figure 4). When added to its higher basal fluorescence and the maintenance of a good selectivity over cAMP (230-fold), these considerations point to H6-FGA^M^ being the leading biosensor. A possible disadvantage compared with the original FlincG, however, is the lack of dimming of the secondary excitation peak at 410 nm in the presence of cGMP (Figure 3B) that could have enabled ratiometric measurements (Nausch et al., 2008).

Beyond their behavior in model HEK293T cells, the key issue is the applicability of the biosensors to other cell types, including neurones. In neuroblastoma cells, the time-courses of responses to exogenous NO and acetylcholine reported by FGB were closely comparable to the dynamics of cGMP measured using a patch-cramming technique (Trivedi and Kramer, 1998, 2002). The observed saturation of biosensor responses at the higher NO concentrations (1 nM and above) is also consistent with cGMP attaining the low micromolar concentrations that were recorded using patch-cramming in response to an NO-donor, although the NO concentrations in those experiments were unknown. The similarities give confidence that the biosensor is faithfully reporting changes in cGMP concentration. The successful expression of functionally-active genetically-encoded biosensors (FGB or H6-FGA^M^) in primary cultures of hippocampal and DRG cells with sufficient brightness to enable imaging of small subcellular domains, opens the way to investigate cGMP-mediated signal transduction in native cells *in vitro* and potentially *in vivo*, with unsurpassed spatial and temporal resolution.

Finally, we would like to propose a simplified FlincG nomenclature (also adopted in the Abstract of this report). The originally published δ-FlincG sequence with part of PKG as the C-terminal tail and no mutations in the cpEGFP domain (Figure 1b of Nausch et al., 2008) would be FlincG1. The biosensor actually used in that same study, however, appears to be most similar to FGB with an arginine-to-cysteine mutation in the cpEGFP domain. We propose that FGB be called FlincG2 and that H6-FGA^M^ becomes FlincG3.

### Conflict of interest statement

The authors declare that the research was conducted in the absence of any commercial or financial relationships that could be construed as a potential conflict of interest.
