# Supplementary material for: Improved genetically-encoded, FlincG-type fluorescent biosensors for neural cGMP imaging
Source: Front Mol Neurosci. 2013 Sep 24;6:26. doi: 10.3389/fnmol.2013.00026 (PMC3781335; doi:10.3389/fnmol.2013.00026)
Supplement: Supplementary file 1 [file DataSheet1.PDF]

Supplementary Material

Improved genetically-encoded fluorescent biosensors for neural cGMP imaging

Yogesh Bhargava, Kathryn Hampden-Smith, Konstantina Chachlaki, Katherine Wood, Jeffrey Vernon, Charles K. Allerston, Andrew M. Batchelor, John Garthwaite

Supplementary Methods. **Analysis of FACS spectra using Mathcad.**

Figure S1. **Multiple protein sequence alignment of FlincG variants.**

Table S1. **Oligonucleotides used in generating and sequencing FlincG clones.**

Supplementary Methods

Analysis of FACS spectra using Mathcad

Experimental data table

Set x-axis range

$x := -0.5, -0.49 .. 4$

data :=

	0	1
0	$7.83\cdot10^{-3}$	46
1	0.016	...

Initial guess values for parameters

$xc := 0.2$ $\quad A := 100$ $\quad w1 := 0$ $\quad w2 := 0.1$ $\quad w3 := 0.1$ $\quad xca := 2$ $\quad Aa := 100$ $\quad w1a := 1$ $\quad w2a := 0.5$ $\quad w3a := 0.2$

$Y := data^{\langle 1\rangle}$ $\quad X := data^{\langle 0\rangle}$

Equation setting the y-value to equal the sum of two asymmetric double sigmoids, as a function of the parameters

$$y(X,xc,A,w1,w2,w3,xca,Aa,w1a,w2a,w3a) := \left[A\cdot\left[\frac{1}{1+e^{-\frac{\left(X-xc+\frac{w1}{2}\right)}{w2}}}\right]\cdot\left[1-\frac{1}{1+e^{-\frac{\left(X-xc-\frac{w1}{2}\right)}{w3}}}\right]+Aa\cdot\left[\frac{1}{1+e^{-\frac{\left(X-xca+\frac{w1a}{2}\right)}{w2a}}}\right]\cdot\left[1-\frac{1}{1+e^{-\frac{\left(X-xca-\frac{w1a}{2}\right)}{w3a}}}\right]\right]$$

Plot of raw data and curve based on guess parameters

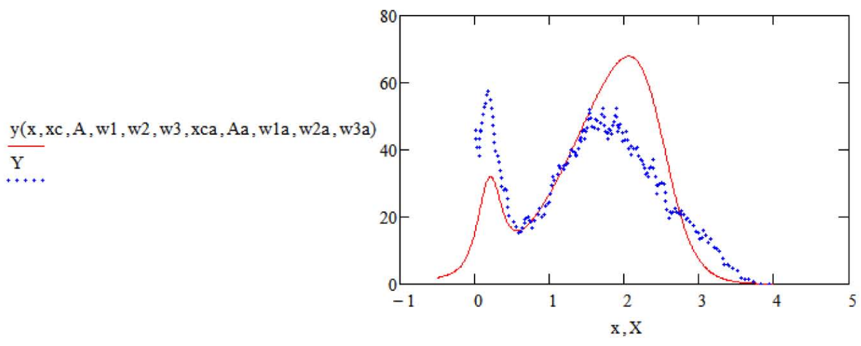

$n := length(Y) - 1$

$n = 510$

$i := 1 .. n$

$CTOL := 10^{-7}$ $\quad TOL := 10^{-7}$ Sets tolerances higher than default (10-3)

$$SSE(xc,A,w1,w2,w3,xca,Aa,w1a,w2a,w3a) := \sum_{i=1}^{n}\left(Y_i - y(X_i,xc,A,w1,w2,w3,xca,Aa,w1a,w2a,w3a)\right)^2$$

Defines sum of the squares of the errors (SSE)

Given

$SSE(xc,A,w1,w2,w3,xca,Aa,w1a,w2a,w3a) = 0$

$y(-2,xc,A,w1,w2,w3,xca,Aa,w1a,w2a,w3a) < 0.0001$

$0 < xc < 1$

$A > 0$

Constraints. The first (SSE = 0) is the most important. The others preclude the occasional aberrations (e.g. using negative peaks or fitting to noise).

Optimize parameters to minimize errors between data and fit

$$\begin{pmatrix} xc \\ A \\ w1 \\ w2 \\ w3 \\ xca \\ Aa \\ w1a \\ w2a \\ w3a \end{pmatrix} := Minerr(xc,A,w1,w2,w3,xca,Aa,w1a,w2a,w3a)$$

Computed best-fit parameters

		0
xc	0	0.143
A	1	147.991
w1	2	0.066
w2	3	0.098
w3	4	0.118
xca	5	1.707
Aa	6	89.773
w1a	7	0.868
w2a	8	0.382
w3a	9	0.493

Value of SSE (obtained two ways) $\quad \frac{ERR}{n-2} = 4.83$ $\quad \frac{SSE(xc,A,w1,w2,w3,xca,Aa,w1a,w2a,w3a)}{n-2} = 4.83$

$$sum(x) := \left[A\cdot\left[\frac{1}{1+e^{-\frac{\left(x-xc+\frac{w1}{2}\right)}{w2}}}\right]\cdot\left[1-\frac{1}{1+e^{-\frac{\left(x-xc-\frac{w1}{2}\right)}{w3}}}\right]+Aa\cdot\left[\frac{1}{1+e^{-\frac{\left(x-xca+\frac{w1a}{2}\right)}{w2a}}}\right]\cdot\left[1-\frac{1}{1+e^{-\frac{\left(x-xca-\frac{w1a}{2}\right)}{w3a}}}\right]\right]$$

Fit using optimized parameters

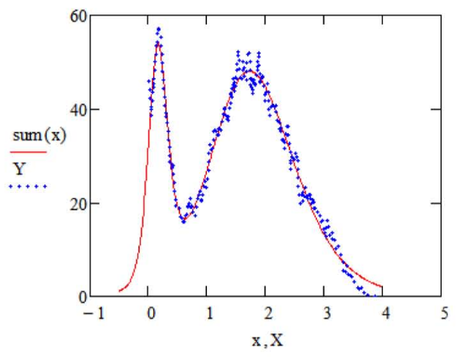

$$untrans(X) := \left[A\cdot\left[\frac{1}{1+e^{-\frac{\left(X-xc+\frac{w1}{2}\right)}{w2}}}\right]\cdot\left[1-\frac{1}{1+e^{-\frac{\left(X-xc-\frac{w1}{2}\right)}{w3}}}\right]\right]$$

Untransfected component in isolation

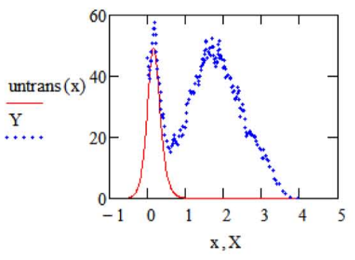

Untransfected component subtracted from data

$subt := \overrightarrow{Y - untrans(X)}$

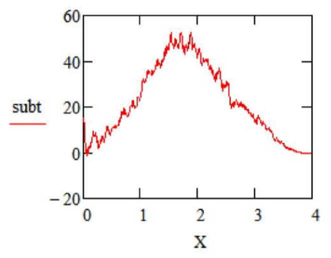

Transfected component in isolation

$$trans(X) := \left[Aa\cdot\left[\frac{1}{1+e^{-\frac{\left(X-xca+\frac{w1a}{2}\right)}{w2a}}}\right]\cdot\left[1-\frac{1}{1+e^{-\frac{\left(X-xca-\frac{w1a}{2}\right)}{w3a}}}\right]\right]$$

Transfected component subtracted from data

$subt1 := \overrightarrow{Y - trans(X)}$

Data plus fit and underlying distributions

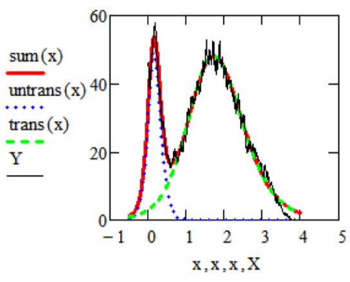

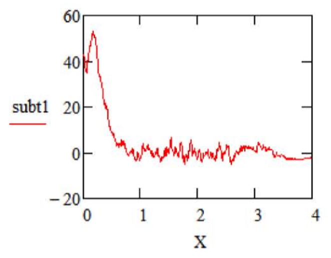

Integrate distributions to find areas $\quad int := \int_{-4}^{4} trans(x)\,dx$ $\quad int1 := \int_{-4}^{4} untrans(x)\,dx$

$int = 89.547$ $\quad int1 = 21.46$

% transfected $\quad \frac{int\cdot100}{int + int1} = 80.668$

Find peak of untransfected profile

$x := -1, -0.99 .. 2$

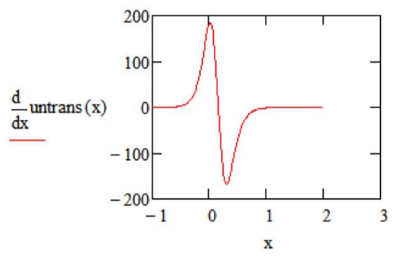

$Peak := root\left(\frac{d}{dx}untrans(x), x, 0, 1\right)$

$Peak = 0.158$

Find median values of distributions

$med := 2$

Given

$$\frac{int}{2} = \int_{-3}^{med} trans(x)\,dx$$

$median := Find(med)$

$median = 1.781$

$10^{median} = 60.413$

$med1 := 0.2$

Given

$$\frac{int1}{2} = \int_{-3}^{med1} untrans(x)\,dx$$

$median1 := Find(med1)$

$median1 = 0.167$

$10^{median1} = 1.467$

```
           1
AdV-DNA    ---------- ---------- ---------- ---------- --------MA LRQAFRKFTK SERSKDLIKE
FGB        ---------- ---------- ---------- ---------- --------MA LRQAFRKFTK SERSKDLIKE
FG^MT      ---------- ---------- ---------- ---------- --------MV LRQAFRKFTK SERSKDLIKE
FGA^M      ---------- ---------- ---------- ---------- --------MV LRQAFRKFTK SERSKDLIKE
H6-FGA^M   MAHHHHHHSS GKETAAAKFE RQHMDSPPPS GLVPRGSAGS GTIDDDDKMV LRQAFRKFTK SERSKDLIKE

          71
AdV-DNA    AILDNDFMKN LELSQIQEIV DCMYPVEYGK DSCIIKEGDV GSLVYVMEDG KVEVTKEGVK LCTMGPGKVF
FGB        AILDNDFMKN LELSQIQEIV DCMYPVEYGK DSCIIKEGDV GSLVYVMEDG KVEVTKEGVK LCTMGPGKVF
FG^MT      AILDNDFMKN LELSQIQEIV DCMYPVEYGK DSCIIKEGDV GSLVYVMEDG KVEVTKEGVK LCTMGPGKVF
FGA^M      AILDNDFMKN LELSQIQEIV DCMYPVEYGK DSCIIKEGDV GSLVYVMEDG KVEVTKEGVK LCTMGPGKVF
H6-FGA^M   AILDNDFMKN LELSQIQEIV DCMYPVEYGK DSCIIKEGDV GSLVYVMEDG KVEVTKEGVK LCTMGPGKVF

         141
AdV-DNA    GELAILYNCT RTATVKTLVN VKLWAIDRQC FQTIMMRTGL IKHTEYMEFL KSVPTFQSLP EEILSKLADV
FGB        GELAILYNCT RTATVKTLVN VKLWAIDRQC FQTIMMRTGL IKHTEYMEFL KSVPTFQSLP EEILSKLADV
FG^MT      GELAILYNCT RTATVKTLVN VKLWAIDRQC FQTIMMRTGL IKHTEYMEFL KSVPTFQSLP EEILSKLADV
FGA^M      GELAILYNCT RTATVKTLVN VKLWAIDRQC FQTIMMRTGL IKHTEYMEFL KSVPTFQSLP EEILSKLADV
H6-FGA^M   GELAILYNCT RTATVKTLVN VKLWAIDRQC FQTIMMRTGL IKHTEYMEFL KSVPTFQSLP EEILSKLADV

         211
AdV-DNA    LEETHYENGE YIIRQGARGD TFFIISKGKV NVTREDSPNE DPVFLRTLGK GDWFGEKALQ GEDVRTANVI
FGB        LEETHYENGE YIIRQGARGD TFFIISKGKV NVTREDSPNE DPVFLRTLGK GDWFGEKALQ GEDVRTANVI
FG^MT      LEETHYENGE YIIRQGARGD TFFIISKGKV NVTREDSPNE DPVFLRTLGK GDWFGEKALQ GEDVRTANVI
FGA^M      LEETHYENGE YIIRQGARGD TFFIISKGKV NVTREDSPNE DPVFLRTLGK GDWFGEKALQ GEDVRTANVI
H6-FGA^M   LEETHYENGE YIIRQGARGD TFFIISKGKV NVTREDSPNE DPVFLRTLGK GDWFGEKALQ GEDVRTANVI

         281
AdV-DNA    AAEAVTCLVI DRDSFKHLIG GLDDVSNKAY EDAEAKAKYE AEAAFFANLK NVYIMADKQK NGIKANFKIR
FGB        AAEAVTCLVI DRDSFKHLIG GLDDVSNKAY EDAEAKAKYE AEAAFFANLK NVYIMADKQK NGIKANFKIR
FG^MT      AAEAVTCLVI DRDSFKHLIG GLDDVSNKAY EDAEAKAKYE AEAAFFANLK NVYIKADKQK NGIKANFKIR
FGA^M      AAEAVTCLVI DRDSFKHLIG GLDDVSNKAY EDAEAKAKYE AEAAFFANLK NVYIKADKQK NGIKANFKIR
H6-FGA^M   AAEAVTCLVI DRDSFKHLIG GLDDVSNKAY EDAEAKAKYE AEAAFFANLK NVYIKADKQK NGIKANFKIR

         351
AdV-DNA    HNIEDGGVQL AYHYQQNTPI GDGPVLLPDN HYLSTQSKLS KDPNEKCDHM VLLEFVTAAG ITLGMDELYK
FGB        HNIEDGGVQL AYHYQQNTPI GDGPVLLPDN HYLSTQSKLS KDPNEKRDHM VLLEFVTAAG ITLGMDELYK
FG^MT      HNIEDGGVQL AYHYQQNTPI GDGPVLLPDN HYLSVQSKLS KDPNEKRDHM VLLEFVTAAG ITLGMDELYK
FGA^M      HNIEDGGVQL AYHYQQNTPI GDGPVLLPDN HYLSTQSKLS KDPNEKRDHM VLLEFVTAAG ITLGMDELYK
H6-FGA^M   HNIEDGGVQL AYHYQQNTPI GDGPVLLPDN HYLSTQSKLS KDPNEKRDHM VLLEFVTAAG ITLGMDELYK

         421
AdV-DNA    GGTGGSMVSK GEELFTGVVP ILVELDGDVN GHKFSVSGEG EGDATYGKLT LKFICTTGKL PVPWPTLVTT
FGB        GGTGGSMVSK GEELFTGVVP ILVELDGDVN GHKFSVSGEG EGDATYGKLT LKFICTTGKL PVPWPTLVTT
FG^MT      GGTGGSMVSK GEELFTGVVP ILVELDGDVN GHKFSVSGEG EGDATYGKLT LKFICTTGKL PVPWPTLVTT
FGA^M      GGTGGSMVSK GEELFTGVVP ILVELDGDVN GHKFSVSGEG EGDATYGKLT LKFICTTGKL PVPWPTLVTT
H6-FGA^M   GGTGGSMVSK GEELFTGVVP ILVELDGDVN GHKFSVSGEG EGDATYGKLT LKFICTTGKL PVPWPTLVTT

         491
AdV-DNA    LTYGVQCFSR YPDHMKQHDF FKSAMPEGYI QERTIFFKDD GNYKTRAEVK FEGDTLVNRI ELKGIDFKED
FGB        LTYGVQCFSR YPDHMKQHDF FKSAMPEGYI QERTIFFKDD GNYKTRAEVK FEGDTLVNRI ELKGIDFKED
FG^MT      LTYGVQCFSR YPDHMKQHDF FKSAMPEGYI QERTIFFKDD GNYKTRAEVK FEGDTLVNRI ELKGIDFKED
FGA^M      LTYGVQCFSR YPDHMKQHDF FKSAMPEGYI QERTIFFKDD GNYKTRAEVK FEGDTLVNRI ELKGIDFKED
H6-FGA^M   LTYGVQCFSR YPDHMKQHDF FKSAMPEGYI QERTIFFKDD GNYKTRAEVK FEGDTLVNRI ELKGIDFKED

         561
AdV-DNA    GNILGHKLEY NLSDLTSLTP LELEVSDE
FGB        GNILGHKLEY NLSDLTSLTP LELEVSDE
FG^MT      GNILGHKLEY N--------- --------
FGA^M      GNILGHKLEY NLSDFNIIDT LGVGGFGR
H6-FGA^M   GNILGHKLEY NLSDFNIIDT LGVGGFGR
```

Figure S1. Multiple protein sequence alignment of FlincG variants. Coloured lines denote different domains, coded as in Figure 1. Shaded regions represent modifications.

Table S1. Oligonucleotides used in generating and sequencing FlincG clones

Primer Name	Sequence (5' to 3')	Remark
PreF	CGCCACCATGG*T*ACTCCGACAGG	Kozak site, alanine to valine mutation
FinF	ACGCTACCGGTCGCCACCATGG*T*ACTCC	AgeI cleavage site
BRPstop	TTCCTCGAG*TTA*GTTGTACTCCAGCTTGTGC	XhoI cleavage site; stop codon
C349R*	CCAACGAGAAG*AGA*GATCACATGGTCCTG	cpEGFP region
M335K*	ACGTCTATATC*AAG*GCCGACAAGCAGAAGAA	cpEGFP region
T385V*	CCACTACCTGAGC*GTC*CAGTCCAAACTTTC	cpEGFP region
Fwd-PBEx	GATGGTACTCCGACAGGCATTCCG	In the pTriEx-4 vector, the first G makes the last base of a lysine codon, forming an enterokinase cleavage site
Rev-MCW-NotEx	TTTGCGGCCGCCTATCGTCCGAAACCTCC	Not I cleavage site
Rev-CR4-NotEx	TTTGCGGCCGCCTACTCGTCCGAAACCTC	Not I cleavage site
New-KH6 1680 f	AGAGCTAGCGACTGGATCCGGACCATGGCACT	To generate C-terminal hexa-histidine tag with carboxypeptidase site
New KH6.1 R	ATGATGATGATGCTTCTCGTCCGAAACCT	To generate C-terminal hexa-histidine tag with carboxypeptidase site
New KH6.2 R	CTCTCTCGAGATTAATGATGATGATGATGATGCTT	To generate C-terminal hexa-histidine tag with carboxypeptidase site
New KX R	CTCTCTCGAGATTACTTCTCGTCCGAAACCT	To mimic carboxypeptidase-cleaved product by adding a C-terminal lysine
sP-Pre-p10	CCGGAGTTAATCCGGGACCT	DNA sequencing for pTriEx-4 vector clones.
sP502-C1	CAACTCCGCCCCATTGACGC	DNA sequencing for C1 vector clones
sP911-PKGC1	ACTGTACCCGGACGGCGACC	DNA sequencing for PKG region.
sP1562-cpGreen	CGGCGACGGCCCCGTGCTGC	DNA sequencing for cpEGFP region.
sP2054-cpGreen	AGACCCGCGCCGAGGTGAAG	DNA sequencing for the tail region.

*Numbering as in Figure S1.
